# Latest Review Papers in Molecular Microbiology

**DOI:** 10.3390/ijms241813990

**Published:** 2023-09-12

**Authors:** Andreas Burkovski

**Affiliations:** Microbiology Division, Friedrich-Alexander-Universität Erlangen-Nürnberg, 91058 Erlangen, Germany; andreas.burkovski@fau.de; Tel.: +49-91318528086

This Special Issue—dedicated to high-quality review papers in molecular microbiology—is highlighting two important developments in the field: (i) the analysis of microbiome data in health and disease and (ii) the search for strategies against bacteria showing antimicrobial resistance.

While formerly studies of microbes and viruses were mainly focused on the isolation, identification, and characterization of especially pathogenic species, the development of next generation sequencing—together with appropriate bioinformatics tools—allows a much more holistic view of the human microbiome. Due to the fact that the intestinal microbiota helps to digest food, supports nutrient uptake, and contributes to the supply of vitamins, the gut microbiome gained early interest, not only in the scientific community but also in the public. Moreover, it was shown that intestinal microbes are crucial for developmental processes such as the maturation of the immune system.

The effect of intestinal microbiota, intraluminal metabolites, and signaling molecules on the development and maintenance of distant tissues, especially on bone physiology, is summarized and discussed in the review by Grüner et al. [1]. The authors describe how the intestinal microbiome can influence bone density, bone remodeling, and postnatal skeletal development. These effects are especially well-studied in the case of inflammatory bowel disease (IBD) and are based on alterations in uptake of, e.g., calcium, magnesium, phosphate, iron, and vitamin D, as well as metabolism and immune functions. Frequently observed extra-intestinal manifestations of IBD caused by dysbiosis in the intestine affect bones (e.g., osteoporosis, osteomalacia) and joints (e.g., arthritis, dactylitis). Obviously, the intestinal microbiome is crucial for the maintenance of gut-bone physiology.

The influence of intestinal microbiota on the peripheral nervous system is summarized by Calabrò et al. [2]. As described in this paper, the gut microbiota influences the regeneration of nerve injuries as well as the function of the autonomic nervous system and the skeletal muscle. Several examples demonstrating the influence of the gut microbiome on pathological states of the nervous system are presented, including neuropathic pain, autism spectrum disorders, autoimmune disorders, and myopathies.

A review focusing on the human male genital tract microbiota is presented by Zuber et al. [3]. The review summarizes studies on the profiling of microbial colonization of different parts of the male genital tract. Moreover, methodological problems and the impact of studies on the human male genital tract on male infertility and pathophysiology are discussed. Interestingly, bacterial abundance seems to be highly variable and range from considerable colonization to almost sterile conditions.

Also, blood is generally thought to be sterile. Exceptions are systemic infections such as bacteremia or septicemia. Cheng et al. [4] present current evidence on the presence of microbial material in the blood stream, controversies in the field, and challenges of blood microbiome studies. The review summarizes the current knowledge about blood microbiota in health and disease. The influence of the microbiota on cardiometabolic and respiratory diseases, cancer, kidney dysfunction, pregnancy complications, and others are discussed. Moreover, controversies, knowledge gaps, and future directions are presented.

Two review papers address new strategies to overcome antimicrobial resistance of pathogenic bacteria. Today, bacterial resistance to antibiotics is a significant global health problem, which is estimated to become an even more severe threat in the next decades. Therefore, new strategies to combat strains resistant to common antimicrobials are urgently needed.

Tabcheh et al. [5] present a new strategy to circumvent (multi)drug-resistance in Gram-negative bacteria. As an alternative to the development of new antibiotics, the latest strategies to rejuvenate common antibiotics and restore their biological efficacy are discussed. The authors present common mechanisms of antibiotic resistance such as membrane impermeability, antibiotic target modification, antibiotic inactivation, and efflux pumps, and strategies to circumvent these by combinations of drugs, drug–enzyme combinations, efflux pump inhibitors, and others.

Máslanka and Mucha present an alternative to established antibiotics: the organoselenium compound ebselen [6]. Besides its antioxidative, anti-inflammatory, and cytoprotective activities, ebselen has antibacterial properties, which are discussed with respect to the current state of research, molecular mechanism of antimicrobial activity, and therapeutic implications.

Taken together, the Special Issue “Latest Review Papers in Molecular Microbiology” provides an excellent overview about the latest developments in microbiota–host interaction and strategies to cope with bacteria developing resistance against common antibiotics and emphasizes the scientific importance of these fields of research at the interface of molecular biology and medicine.
